# Interindividual differences in environmentally relevant positive trait affect impacts sustainable behavior in everyday life

**DOI:** 10.1038/s41598-021-99438-y

**Published:** 2021-10-14

**Authors:** Kimberly C. Doell, Beatrice Conte, Tobias Brosch

**Affiliations:** Department of Psychology and Swiss Center for Affective Sciences, University of Geneva, Geneva, Switzerland

**Keywords:** Psychology, Human behaviour

## Abstract

Emotions are powerful drivers of human behavior that may make people aware of the urgency to act to mitigate climate change and provide a motivational basis to engage in sustainable action. However, attempts to leverage emotions via climate communications have yielded unsatisfactory results, with many interventions failing to produce the desired behaviors. It is important to understand the underlying affective mechanisms when designing communications, rather than treating emotions as simple behavioral levers that directly impact behavior. Across two field experiments, we show that individual predispositions to experience positive emotions in an environmental context (trait affect) predict pro-environmental actions and corresponding shifts in affective states (towards personal as well as witnessed pro-environmental actions). Moreover, trait affect predicts the individual behavioral impact of positively valenced emotion-based intervention strategies from environmental messages. These findings have important implications for the targeted design of affect-based interventions aiming to promote sustainable behavior and may be of interest within other domains that utilize similar intervention strategies (e.g., within the health domain).

## Introduction

Emotions exert a powerful influence on human behavior. They help us detect and understand risks and opportunities, signal that something that is important for our concerns and values is being threatened or supported, and they drive our actions by providing the necessary motivational momentum^1–3^. In the context of climate change and sustainable action, emotions can fulfill these functions by making people aware of the urgency to act to mitigate climate change and by providing a motivational basis to engage in sustainable action.

Accumulating research demonstrates how the experience of both emotions and affect influence the willingness to act to promote sustainability. Emotions are generally defined as adaptive reactions that are elicited when an event or object is appraised as relevant to one’s concerns, which often considerably impact subsequent decision-making and behavior; whereas affect is generally a more subtle positive/negative feeling experienced towards an event which can more subtly inform decisions and judgments^1,3,4^. Together affect and emotional responses are some of the strongest predictors when it comes to predicting a variety of climate change related judgements and behaviors (e.g., risk perceptions, willingness to act, etc.)^4–6^.

Overall, people who experience strong affective/emotional reactions toward climate change judge the related risks to be higher and are willing to alter their behavior to a larger extent^7–11^. Individual differences in the extent to which people report experiencing specific emotions such as worry, distress, interest, hope, or pride in the context of climate change have all been associated with the willingness to take up mitigation actions^12–15^. Interestingly, such effects have not only been shown for emotions that people actually experience, but also for the emotions people *expect* to experience^1,16–19^. The anticipation of positive affective reactions can directly motivate sustainable or pro-social behavior when the behavior is expected to be experienced as hedonically pleasurable or morally rewarding; a phenomenon known as “warm glow”^20,21^. Conversely, a person may avoid specific behaviors because they anticipate negative affective reactions; a phenomenon known as “a cold prickle”^22^. Anticipated affective reactions have been shown to be important predictors of a range of sustainable behaviors, including various transportation, recycling, and energy-saving behaviors^15,23–25^. Thus, both experienced and anticipated emotions may operate as drivers of sustainable behavior.

### Difficulties with leveraging emotions to promote sustainable behavior

Given their enormous potential, it is only logical to try to leverage affect and emotion in an attempt to promote sustainable actions in experimental set-ups and large-scale emotional climate communications. And indeed, messages aiming to induce emotions such as hope, guilt, or anger, have in some cases led to more sustainable intentions and behaviors^12,26,27^. However, emotion-eliciting messages and interventions have not always been effective, as other studies either failed to produce the desired results or even yielded opposing, unintended boomerang effects. For instance, messages designed to induce hope or general positive affective states have been found to increase willingness to act, to have no effect at all, to *decrease* climate change risk perception, and, in some cases, to even induce anger and resentment^12,28–31^. Similarly, messages designed to induce fear of climate change have been found to increase willingness to act, to have no effect, and to reduce peoples’ perceived response efficacy^32–34^. Thus, while the experience or the anticipation of “naturally occurring” state emotions or warm glow have been shown to relate to more sustainable behavior, inducing them does not necessarily lead to the same results. As argued previously^35^, emotions are not simple levers that one can pull in order to promote a desired behavior, and treating them as such often does not work or sometimes even leads to boomerang effects that reduce sustainable behavior^36^.

### Individual differences concerning the elicitation of environmental emotions

We argue here that it is important to consider the inter-individual differences in the mechanisms underlying affect and emotions in the context of sustainable action. Not everyone will experience the same “amount” of emotion, or even the same discrete emotion when encountering a specific stimulus or situation. This tendency to experience such emotions in a predetermined manner (i.e., as a result of being exposed to specific types of stimuli) is called “trait affect”, and changes from individual to individual. One’s concerns, experiences, and values will impact the way that a stimulus is interpreted, and thus influence which emotions are elicited, and to what extent^8,37,38^. However, attempts to manipulate and induce emotions towards an issue or a behavior which are incompatible with a person’s concern structure may be ineffective or may even boomerang and produce reactance^39^.

It is thus important to consider individual predispositions to experience emotions, i.e., trait affect, in relation to a given topic or behavior. A recently developed measure of environmentally relevant *positive trait affect* (from here on referred to as “trait affect”) assesses an individual’s predisposition to experience positive emotions in situations with positive environmental outcomes^8^. Using items such as “I feel proud when I act in an environmentally friendly manner” and “I feel appreciation towards others when they act in an environmentally friendly manner”, this instrument measures to what extent an individual usually experiences positive affect from their own actions as well as when witnessing other people’s positive behaviors. It moreover reflects the anticipation of positive affect in an environmental context, as a person who in the past has often felt proud after showing pro-environmental behavior is more likely to expect this emotion in the future. Importantly, individual trait affect predicted the impact of an emotion induction on sustainable behaviors in a laboratory-based social dilemma task^40^: Guilt and pride inductions increased sustainable actions via reductions in consumption and increases in investments, respectively, but only in participants with high levels of trait affect.

### Investigating the impact of affect on sustainable behavior in real life

While these results are promising, in order to better understand the dynamics of affect and emotions underlying sustainable behaviors, more research needs to be conducted via real-world field-based experiments. The majority of previous studies have utilized either lab-based methodologies, where individuals react to hypothetical situations, or survey-based approaches, where individuals retrospectively report about their past behavior. Although these experiments provide valuable insights, it is not entirely clear how their results map onto everyday occurrences of environmental behaviors and their emotional antecedents and consequences, as emotions are transient phenomena that change dynamically and continuously throughout the day^41^. Retrospective self-reports may moreover be influenced by memory biases driven by consistency or social desirability aspects which may potentially inflate correlations between measures of emotions and behavior^42^.

Here, across two large experiments, we use experience sampling, a field-based methodology where participants were asked to report various environmental actions and their subsequent affective state multiple times a day for several days. Here, we aimed to better understand how interindividual differences in (environmentally relevant) positive trait affect impact both sustainable behaviors, and subsequently experienced affective state. More specifically, in Experiment 1 we show that people with high levels of positive trait affect (i.e., people who tend to experience strong positive emotions in situations with positive environmental outcomes) commit more pro-environmental actions and report greater shifts in subsequent positive affective states. These affective shifts were observed both for pro-environmental actions committed personally and for pro-environmental actions that participants were exposed to by others (i.e., observed first-hand or learned about). These results provide first evidence that changes in affective states may also occur vicariously, when being exposed to the “good” actions of others. Based on these findings, in Experiment 2 we aimed to induce vicarious affective states via positive and negative environmental messages, in order to more causally determine how such stimuli impact environmental behavior as a function of positive trait affect. We observed that in people with high levels of positive trait affect, being exposed to positive environmental messages in the morning increased subsequently experienced positive emotions and resulted in the commission of more pro-environmental actions throughout the rest of the day. However, in a subset of people with low levels of positive trait affect the same exposure resulted in the commission of *fewer* pro-environmental actions throughout the rest of the day, providing evidence of a message-induced boomerang effect.

## Results

### Experiment 1: the impact of positive trait affect on environmental behavior and experienced affect in real life

We utilized a novel ecological experience sampling paradigm in which 181 participants (aged 18 to 76, mean = 33.5; 61% female) reported several times per day to what extent they had recently performed or were exposed to environmentally relevant behaviors (ERBs) as well as their current affective state. Participants received messages on their smartphone five times a day for ten days and were asked to report an environmental behavior that occurred within the last hour. They provided a brief description of the behavior and categorized it according to whether it was a *committed positive ERB* (i.e., with a positive impact on the environment), *committed negative ERB*, *exposed to* (i.e., seen, read or heard about) *positive ERB*, *exposed to negative ERB*, or *nonERB* (in case they did not experience an ERB during the relevant interval, they were asked to report a non-environmental behavior). Table 1 illustrates some of the behavioral descriptions provided by participants. Finally, participants reported the valence of their current affective state (from 0 = very negative to 10 = very positive). During participant intake, after providing informed consent, positive trait affect (from the positive outcome affect subscale of the Environmental Trait Affect Questionnaire^8^), value orientations (using a combination of the Schwartz and Steg Value Scales^43–45^), Social Desirability Scale^46^, and demographic information were assessed.

**Table 1:** Examples of verbatim participant responses by category.

	Committed positive	Committed negative	Exposed to positive	Exposed to negative	NonERB
Experiment 1	I recycled my bottle Bought a solar panel and inverter for my house I took a quick shower	Used plastic bags for garbage Burning wood Used energy to watch tv	I saw some people driving Tesla Watched an interview on tv about carbon credits	Saw somebody throw rubbish from their car Watched a documentary about water pollution in China	I read a book Sleeping Made fun of someone behind his back
Experiment 2	Wash laundry with cold water I replaced all the lighting in my house with LED bulbs We had totally vegetarian meal	I took an extra long shower to relax I tossed trash on the ground because there was no garbage can near by I threw my cigarette out the car window	Listened to a speech about the green new deal I read an article about a device in the ocean that will suck up garbage Saw a sign at the gym about a recycling program	Trump says windmills cause cancer My dad told me about the coral reefs being destroyed I had seen an video on world’s most polluted river located in Indonesia	Helped my wife with the dishes I told a joke to a friend I danced with my 2 year old

**Figure 1:**
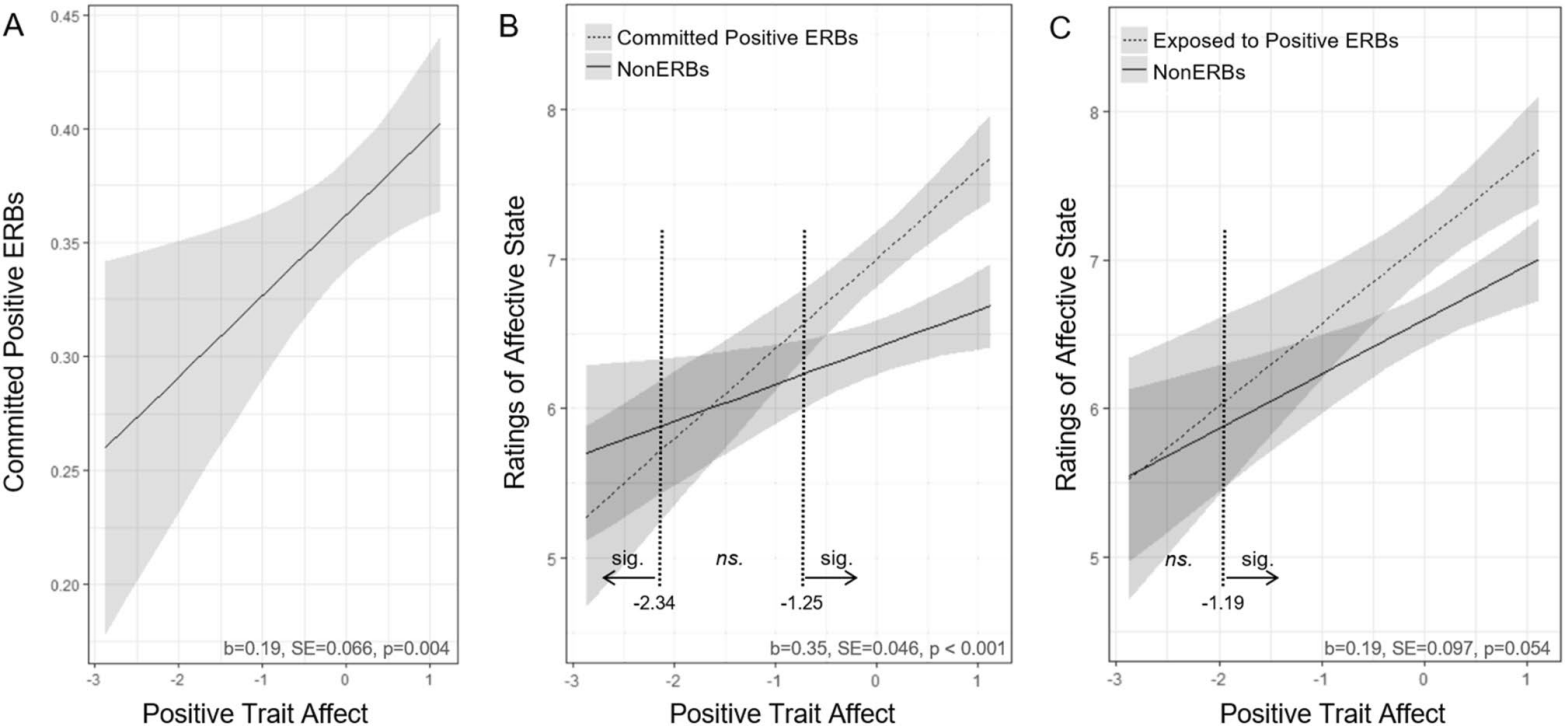
The impact of trait affect on environmental behavior and experienced affect in real life in Experiment 1. (**A**) Line graph illustrating the positive relationship between trait affect and likelihood to commit positive ERBs. (**B**) Interaction between trait affect and committed positive ERBs compared to NonERBs. (**C**) Interaction between trait affect and exposed to positive ERBs compared to NonERBs. Vertical dotted lines illustrate where slopes significantly differ from each other as determined by simple slopes analyses. All graphs show predicted values, the grand mean centered values of trait affect (positive outcome ETA) and 95% confidence intervals as estimated from their respective regression models. ERB = environmentally relevant behavior; NonERBs = behaviors reported that were not environmentally relevant.

In total, participants responded to 7,161 individual signals (mean response rate of 79%). In a first step, we analyzed to what extent individual differences in trait affect were related to the frequency with which participant committed environmental actions. To this end, we conducted two mixed-effects logistic regression analyses with trait affect as independent variables and positive and negative ERBs as dependent variables, respectively. Biospheric and egoistic value orientations as well as key demographic variables were added as covariates (see^8^). Results showed that trait affect was positively associated with an increased likelihood to commit positive ERBs (Fig. 1A; Table 2; OR = 1.21, CI = 1.06–1.38, *p* = 0.004; Tables S2, S3). None of the predictors of interest predicted likelihood to commit negative ERBs (Supplementary Table S3).

**Table 2:** Multilevel binomial logistic (with logit link) regression model predicting likelihood to commit positive environmental behaviors (i.e. positive ERBs) in Experiment 1.

Predictors	Odds ratios	95% CI	*p*
(Intercept)	0.53	0.47–0.60	< 0.001
Positive trait affect	1.21	1.06–1.38	0.004
Biospheric values	1.00	0.91–1.09	0.929
Egoistic values	1.08	0.96–1.22	0.190
Social desirability scale	1.04	1.00–1.07	0.048
Age	1.01	1.00–1.02	0.070
Gender	0.96	0.85–1.07	0.449
Time	0.99	0.99–1.00	< 0.001
**Random effects**			
σ^2^	3.29		
τ_00_	0.55_Participant_		
τ_11_	0.00_Participant. Time_		
ρ_01_	0.82_Participant_		
ICC	0.15		
N	180_Participant_		
Observations	7136		
Marginal R^2^	0.019		
Conditional R^2^	0.164		

For comparative purposes, a similar model without the covariates is shown in Supplementary Table S2.

*ERBS* Environmentally relevant behaviors.

We then analyzed to what extent committing or being exposed to environmental actions was related to participants’ affective state, and to what extent individual differences in positive trait affect moderated the relationship with the positive (committed and exposed to) behaviors. To this end, we conducted a linear mixed-effects model with the different ERB types and trait affect as independent variables and current affective state as the dependent variable (Table 3). To assess the ranges of the significance of the trait affect moderation, we conducted a simple slopes analysis using the Johnson–Neyman technique^47^. Results showed that compared to nonERBs, both committing and being exposed to positive ERBs resulted in a more positive affective state (committed positive ERB: *b* = 0.59, CI = 0.49–0.68, *t*(7002) = 12.2, *p* < 0.001; exposed to positive ERB: *b* = 0.52, CI = 0.35–0.69, *t*(6923) = 5.93, *p* < 0.001), while both committing and being exposed to negative ERBs resulted in a more negative affective state (committed negative ERB: *b* =  − 0.87, CI =  − 0.99 to − 0.75, *t*(6970) =  − 14.1, *p* < 0.001; exposed to negative ERB: *b* =  − 1.53, CI =  − 1.72 to − 1.33, *t*(6985) =  − 15.5, *p* < 0.001). Importantly, individual differences in positive trait affect moderated the effects of the positive behaviors: On the one hand, participants with high levels of trait affect reported positive affective shifts after reporting committing positive ERBs compared to reporting NonERBs (interaction: *b* = 0.35, CI = 0.26–0.44, *t*(6989) = 7.69, *p* < 0.001, Fig. 1B). Participants with low levels of trait affect, on the other hand, reported *negative* affective shifts after reporting committing positive ERBs compared to reporting NonERBs (as shown by the vertical dotted lines in Fig. 1B). In addition, after being exposed to a positive ERB (compared to NonERBs), participants with high levels of trait affect reported (statistically marginally) larger positive affective shifts (interaction: *b* = 0.19, CI = 0.00–0.38, *p* = 0.053, Fig. 1C).

**Table 3:** Multilevel linear regression model predicting state affect in Experiment 1.

Predictor	Standardized estimates	95% CI	*p*
(Intercept)	6.55	6.36–6.74	< 0.001
Committed positive ERBs	0.59	0.49–0.68	< 0.001
Exposed to positive ERBs	0.52	0.35–0.69	< 0.001
Committed negative ERBs	− 0.87	− 0.99 to − 0.75	< 0.001
Exposed to negative ERBs	− 1.53	− 1.72 to − 1.33	< 0.001
Positive trait affect	0.24	0.04–0.43	0.019
Committed positive ERB × Positive trait affect	0.35	0.26–0.44	< 0.001
Exposed to positive ERB × Positive trait affect	0.19	− 0.00–0.38	0.054
Time	− 0.08	− 0.14 to − 0.02	0.008
Social desirability scale	0.07	0.02–0.13	0.011
Age	0.01	− 0.01–0.03	0.209
Gender	− 0.04	− 0.22–0.15	0.691
**Random effects**			
σ^2^	2.52		
τ_00_	1.42_Participant_		
τ_11_	0.09_Participant.time_		
ρ_01_	0.19_Participant_		
ICC	0.37		
N	180_Participant_		
Observations	7130		
Marginal R^2^	0.142		
Conditional R^2^	0.463		

For comparative purposes, a similar model without the covariates, and without the interactions, is shown in Supplementary Table S4.

*ERBS* Environmentally relevant behaviors.

Taken together, Experiment 1 showed that high levels of positive trait affect are associated with (i) a higher number of committed positive ERBs, and (ii) stronger shifts in positive affective state after committing positive ERBs as well as after being exposed to positive ERBs. Additionally, low levels of positive affect were associated with more negative affective shifts after committing positive ERBs. These findings emphasize the importance of interindividual differences in positive affect in the context of real-life sustainable behavior and provide initial evidence for vicariously experienced environmental affect as a potential driver of environmental behavior.

### Experiment 2: trait affect and the impact of vicarious environmental affect on environmental behavior

Here we expanded on the role of vicariously induced affect by investigating whether communications about positive (as compared to neutral and negative) environmental actions can lead to an increase in the commission of positive ERBs, while considering to what extent individual differences in trait affect may constitute a boundary condition for this intervention effect. To this end, 331 participants (aged 18 to 64, mean = 36.2; 48% female) received an environmental message each morning. They were either exposed to news about environmentally positive events (e.g., successful “re-greenification” efforts which planted tens of millions of trees; N = 108), environmentally negative events (e.g., how the last several years have been the hottest in history, leading to hundreds of billions of dollars in damages; N = 108), or non-environmental events (e.g., study results concerning the eating habits of snakes; N = 115). To note, these stimuli were designed to mimic real news headlines/articles that people may likely encounter on a regular basis. After reading the news, participants were subsequently asked to rate the intensity of 4 positive emotions (pride, joy, hope, relief) and 4 negative emotions (anger, disgust, guilt, fear) experienced after reading the information. Participants were contacted three times throughout the rest of the day and asked to report to what extent they had recently performed or been exposed to ERBs, similar to Experiment 1. This protocol lasted for three days in total, resulting in responses to 2203 individual signals (mean response rate of 74%). Participants also completed the experimental intake, including providing informed consent, and completed the same set of questionnaires as in Experiment 1.

We first analyzed whether interindividual differences in trait affect influenced the extent to which participants experienced positive affective shifts after reading the environmental news events. We conducted a multiple linear regression with type of news event and trait affect as predictors and the averaged valence of the four experienced positive emotions as a dependent variable (Cronbach’s alpha = 0.91). Significant interaction effects indicated that participants with higher levels of trait affect experienced higher average levels of positive emotions when exposed to environmentally positive news, as well as lower levels of positive emotions when exposed to environmentally negative news (positive compared to non-environmental news interaction with trait affect: *b* = 6.10, CI = 0.05–0.40, *t*(323) = 2.49, *p* = 0.01; negative compared to non-environmental news interaction with trait affect: *b* =  − 5.88, − 0.41 to − 0.02, *t*(323) =  − 2.17, *p* = 0.031; positive compared to non-environmental news: *b* = 1.20, CI = 1.02–1.38, *p* < 0.001; negative compared to non-environmental news: *b* =  − 0.46, CI =  − 0.64 to − 0.29, *p* < 0.001; Supplementary Table S5).

**Figure 2:**
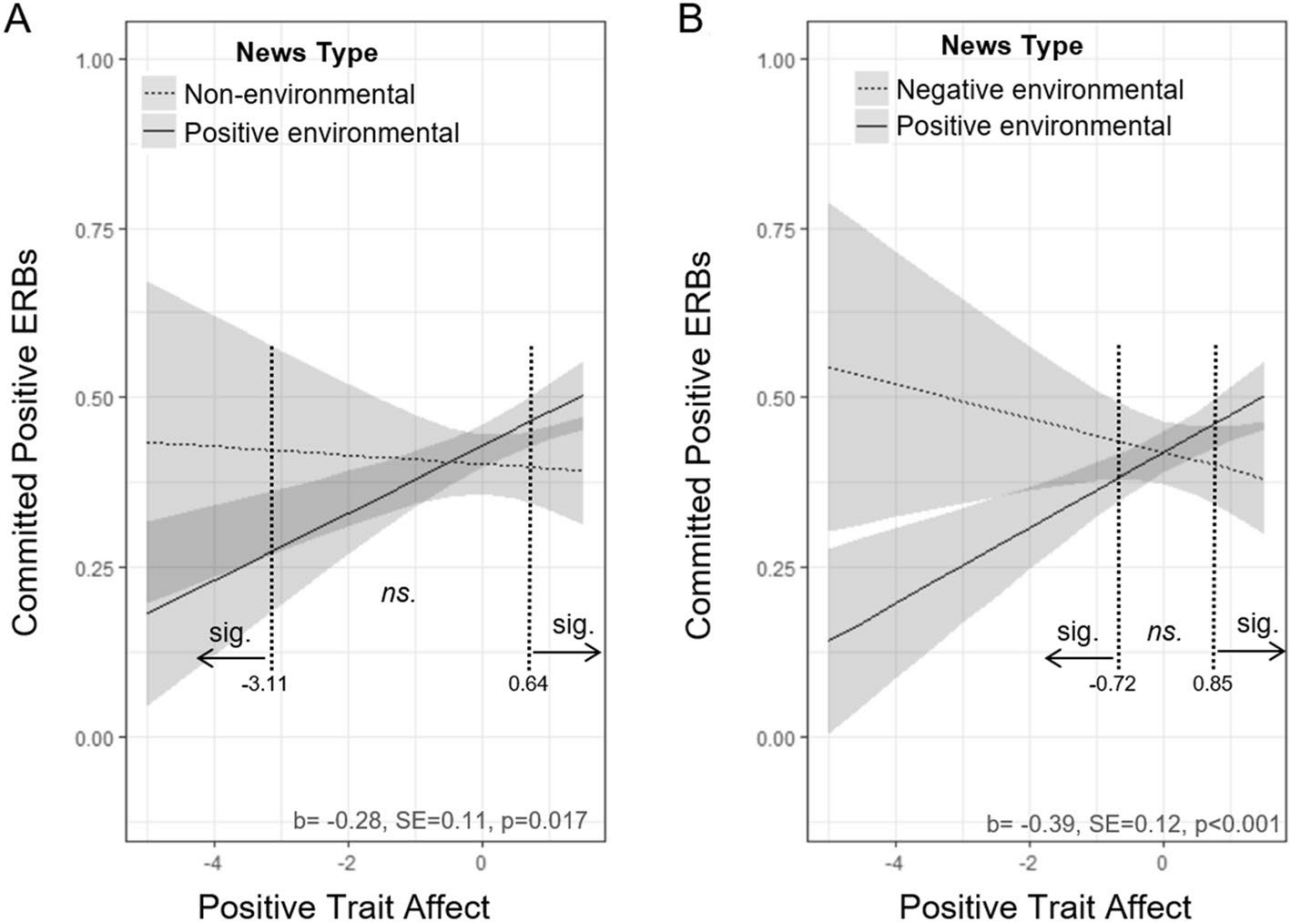
Trait affect, affective environmental news messages, and pro-environmental behaviors in Experiment 2. (**A**) Interaction between trait affect and positive versus non-environmental news messages on committed positive ERBs. Vertical dotted lines illustrate where slopes significantly differ from each other as determined by simple slopes analyses (it should be noted that the lower cutoff at − 3.11 represents the bottom 2.2% of all participants). (**B**) Interaction between trait affect and positive versus negative environmental news messages on committed positive ERBs. Vertical dotted lines again illustrate where slopes significantly differ from each other as determined by simple slopes analyses (it should be noted that the lower cutoff at − 0.72 represents the bottom 18.8% of all participants). All graphs show predicted values, grand mean centered values of trait affect, and 95% confidence intervals as estimated from their respective regression models. ERB = environmentally related behavior.

We next analyzed to what extent exposure to the different types of environmental news had an impact on ERB commission throughout the day, and whether individual trait affect moderated this impact. To this end, we conducted a mixed-effects logistic regression analysis with type of news event and trait affect as predictors and frequency of committed positive ERBs as the dependent variable (Table 4). Significant interactions between news type and trait affect revealed that participants with high trait affect who read a communication about positive environmental news events were more likely to report committing a positive ERB compared to those that read negative environmental news or non-environmental news (see Fig. 2, comparison non-environmental versus positive news OR = 0.75, CI = 0.60–0.95, *p* = 0.01; comparison negative versus positive news: OR = 0.67, CI = 0.54–0.85, *p* < 0.001). A simple slopes analysis using the Johnson–Neyman technique allowed us to determine where the slopes of the different groups significantly differed from each other (dotted vertical lines shown in Fig. 2). Participants with high levels of trait affect who were exposed to positive environmental news in the morning had a higher likelihood to commit positive ERBs throughout the rest of the day (compared to participants exposed to negative or non-environmental news). However, participants with low levels of trait affect who were exposed to positive environmental news in the morning had a *lower* likelihood to commit positive ERBs (again, compared to participants exposed to negative or non-environmental news).

**Table 4:** Multilevel binomial logistic regression (with logit link) results predicting likelihood to commit positive ERBs in Experiment 2.

Predictors	Odds ratios	CI	*p*
(Intercept)	0.72	0.60–0.86	< 0.001
Non-environmental news group	0.90	0.71–1.16	0.429
Negative environmental news group	1.02	0.80–1.31	0.855
Positive trait affect	1.45	1.24–1.69	< 0.001
Non-environmental news group × positive trait affect	0.75	0.60–0.95	0.017
Negative environmental news group × positive trait affect	0.67	0.54–0.85	0.001
Age	1.01	1.00–1.02	0.066
Social desirability scale	1.03	1.00–1.06	0.022
Gender	0.88	0.79–0.97	0.013
Biospheric values	1.12	0.99–1.26	0.070
Egoistic values	1.02	0.91–1.15	0.739
**Random effects**			
σ^2^	3.29		
τ_00 pcpID_	0.17		
ICC	0.05		
N_pcpID_	328		
Observations	2190		
Marginal R^2^	0.040		
Conditional R^2^	0.088		

The grouping variables are effects coded such that the positive environmental news group represents the “baseline”. For comparative purposes, a similar model without the covariates, and without the interactions, is shown in Supplementary Table S4.

*ERBS* Environmentally relevant behaviors.

## Discussion

Across two experiments we show that inter-individual differences in positive trait affect do not only influence the extent to which positive affect is experienced, but also influence the commission of sustainable behaviors in everyday life. These results may help explain why affective messages which aim to promote sustainability may not have the same effect on everyone^12,29–31,48^. Across both experiments, people with high trait affect showed more pronounced positive affective shifts both after committing and after being exposed to positive environmental actions. At the behavioral level, high trait affect was moreover related to the commission of more pro-environmental behaviors in general (Exp. 1) and to committing more pro-environmental behaviors after being exposed to emotion-inducing communications about pro-environmental news items, even hours later (Exp. 2). Low trait affect, on the other hand, was related to decreased positive affective shifts after committing positive ERBs (Exp. 1) and, in a small percentage of participants, even resulted in the commission of *fewer* pro-environmental behaviors after exposure to positive environmental news. These results are consistent with previous findings^12,31,39^ which suggest that in specific participants, manipulations that are incompatible with a person’s affective concern structure may produce reactance, and here, even resulted in an *affective boomerang effect* that decreased sustainable behaviors overall.

These results have important implications for policy makers and communicators, not only in the climate change domain, but across other domains that utilize similar messaging strategies (e.g., health, financial, pollical, etc.). First, they provide evidence that suggests that message tailoring, a strategy that focuses on individual-level characteristics when designing intervention messages^49^, is important to induce a desired behavior change. Second, they point to affective predispositions (e.g., trait affect) as a central characteristic that should be the focus when designing such strategies. Third, utilizing messages that are positively valenced likely efficiently sidesteps multiple potential negative repercussions/concerns that have been raised over the use of negatively valenced messages (e.g., increasing depression/demoralization^35^, drawing from “finite pools of worry”^50^, etc.). Finally, they demonstrate that the tailoring of affective messages (and the evaluation of their outcomes) is possible, even with relatively simple and straightforward manipulations. Predispositions to experience positive emotions in association with pro-environmental behaviors (i.e., a trait “warm glow”) can be leveraged in order to promote sustainability. However, these interventions must take into account that they will not work for everyone, and even may boomerang for individuals with “incompatible” affective predispositions.

There are still multiple important questions that remain, however. For example, what are the long-term effects of such environmental communications that span over weeks or years? It is possible that repeatedly reminding participants via affective messaging may result in “numbness” or potentially alternate boomerang effects that we did not capture here. Additionally, we did not disentangle behaviors with high- and low-environmental impact. While we did capture both types of behavior inside of our datasets (see Table 1), it is possible that there are different affective mechanisms at play when it comes to promoting high- versus low-impact behavior (e.g.,^51^). Another potential limitation is that we cannot verify whether the behaviors reported were real (a relevant issue that is congruent with much of the self-report work in this domain). These points should be a focus for future investigation and considered when designing emotion-based interventions. Regardless, our findings provide real-world empirical support for the notion that emotional “one-size fits all”-styled communication strategies are not optimal for promoting pro-environmentalism^35^.

Our results moreover introduce the distinction between "direct warm glow” based on one’s own positive environmental actions, and a “vicarious warm glow” based on being exposed to others’ positive environmental actions. While Experiment 1 shows that both one’s own and others’ positive environmental actions can result in a positive affective shift, Experiment 2 provides evidence that the vicarious warm glow can be leveraged to promote pro-environmental behaviors, at least in individuals with high levels of trait affect. Humans are an innately social species for whom observational learning powerfully shapes behavior^52^. Previous research has shown that watching someone else obtain a reward can be experienced as rewarding in itself, especially if that person is perceived as being close and personally relevant^53,54^. This reward may motivate further pro-social behaviors^4,54,55^, which in turn result in more (vicariously induced) warm glow^20^, thus initiating a prosocial feedback loop. Thus, one of the most interesting implications of our results may suggest that such a vicarious response loop may extend to concerns of sustainability and sustainable behaviors. Indeed, previous work has shown that concerns and “objects of care” that are threatened by climate change are personally relevant and can produce strong emotional responses^10,50^. Consistent with this, here we illustrate for the first time the role of vicarious emotions as an antecedent to, and a consequence of, pro-environmental behavior.

This pattern of results is also consistent with other relevant theoretical and empirical frameworks from positive and environmental psychology, and the affective sciences. Given the benefits associated with natural environments (e.g., nature exposure increases well-being^56^, restores attention^57^, and conveys health benefits^58^), it is not surprising that people are not only connected to nature, but that they would feel a sense of accomplishment, and other positive emotions (e.g., pride) when acting to protect it. According to the positive affect hypothesis, these positive emotions are evolutionarily adaptive, protect us from a variety of mental disorders, and due to their rewarding/positive nature, lead to more life satisfaction and well-being^59^. This is also well aligned with the Broaden-and-Build theory^60^, which suggests that positive emotions expand people’s thought patterns thus allowing them to consider new and alternative ways of thinking and behaving. Thus, putting together these frameworks in the context of sustainability, positive emotions arise from personal achievements (e.g., behaving pro-environmentally), broadening the scope of behaviors that may lead to similar positive experiences in the future (i.e., increase anticipation of positive affect in the future), ultimately motivating the commission of further similar behaviors^4,15,18,61^. Adding our results to these frameworks, it may suggest that exposure to the good deeds of others results in positive affective shifts (i.e., vicariously induced warm glow), which may act to broaden the scope of pro-environmental behaviors and kick-start this virtuous cycle.

Taken together, our findings suggest that environmental emotions and environmental warm glow can be leveraged via interventions to promote pro-environmental actions. Participants with a predisposition to experience positive affect after pro-environmental actions are more likely to engage in them, and to experience more intense affective shifts afterwards, thus receiving an internal reward for an action that primarily benefits others. This reward in turn validates and reinforces expectations to feel good after future pro-environmental behaviors, triggering a positive feedback loop which may result in further pro-environmental behaviors in the future. Our results moreover suggest the intriguing possibility to trigger this feedback loop via vicarious warm glow elicited by other peoples’ pro-environmental actions One promising future research direction would be to develop strategies to increase to what extent positive emotions are experienced after committing or being exposed to pro-environmental actions. However, these strategies need to be adapted to the context and the target audience and to be empirically tested to ensure that no boomerang effects or other non-intended effects occur.

When interpreting the results of the experiments presented here, one needs to consider the potential impact of the experience sampling methodology as a potential driver of behavior change. One previous experiment utilizing experience sampling in participants who were attempting to quit smoking showed that repeatedly reporting one’s behavior can have a positive cathartic effect on mood and anxiety symptoms, and can ultimately lead to a reduction in cigarette cravings^62^. Similarly, the possibility exists that in our participants the experience sampling directly altered behavior (e.g., “I should ride my bike today so I can report it later and then feel good about it”) or increased awareness about what may not have otherwise been considered as an environmentally relevant behavior (see Table 1). It is however unlikely that these effects had a large confounding impact on the pattern of results related to the link between affect and sustainable action presented above. To go one step further, instead of conceptualizing this aspect as a limitation, it may be reframed as a potential intervention technique, with campaigns that utilize approaches which resemble the experience sampling methodology being an interesting tool to increase self-awareness and promote sustainable actions.

Affective responses play an important role in our reactions to climate change and, if leveraged correctly, may be an important motivating factor to promote sustainable action. By using a real-life experience sampling approach, we were able to gain insights into the interplay of trait affect, experienced affect, and sustainable actions that may not have been possible otherwise. This has yielded important insights about the potential of targeted affective intervention strategies to influence real-world behaviors. Our results help clarify how information campaigns that target positive emotions may have widespread effects on behavior, in both positive and negative directions.

## Methods

### Experiment 1

Participants for Experiment 1 were recruited online between October 2017 and April 2018 in two large-scale advertisement waves via various forums (Facebook groups, crowdsourcing and citizen science webpages, Reddit, and Amazon’s Mechanical Turk). It should be noted that because we used a self-selection convenience sampling procedure, our sample might not be completely representative. To be included in the study, participants were required to be at least 18 years old, have a personal smart phone with an active data plan, successfully answer 3 attention checks in the questionnaires, and respond to at least half (i.e. 25/50) of the experience sampling messages (see the supplementary materials for a detailed description of the data cleaning). During experiment intake, all participants completed the informed consent, provided demographic information (e.g. age, gender), and completed the questionnaires (i.e. Environmental Trait Affect Questionnaire^8^, value orientations (using a combination of the Schwartz and Steg Value Scales^43–45^), and the Social Desirability Scale short form^46^. Finally, participants were required to complete a short training where they received seven examples of different types of environmental behaviors/non-environmental behaviors, and were asked to classify them as a “committed positive environmental behavior”, “committed negative environmental behavior”, “seen/read/heard about positive behavior”, “seen/read/heard about a negative behavior”, or “not environmentally relevant”.

Participants were offered a $10 compensation plus an additional $0.25 for each text message they responded to (maximum of $22.50 USD) in Amazon Online gift cards (or paid via Mechanical Turk). The experience sampling protocol utilized an SMS survey distribution approach, wherein the participants received a hyperlink to a short survey on Qualtrics (Qualtrics.com), via text message, directly to their personal smartphone. Participants were signaled 5 times per day for 10 days randomly between the hours of 9 am and 10 pm. If they did not respond, they were sent a reminder message within 15 min, and after 1 h the signal expired. Participants were first asked to classify their ERB, then give a brief description, and finally rate their current affective state (i.e., mood) on a 11-point scale from “very negative” to “very positive”. Here we report a brief account of the analyses, more detail can be found in the supplementary materials, and in the open-sourced scripts and data which can be found at osf.io/7kmp8. This research was approved by the ethics committee of the Faculty of Psychology and Educational Sciences of the University of Geneva, Switzerland and all research was performed in accordance with relevant guidelines and regulations. Written informed consent was obtained from all participants.

We first analyzed to what extent trait affect differences were related to the frequency with which participants commit positive ERBs. We conducted a mixed effects logistic regression (with logit link; implemented in R with the lme4 package^63^), which predicts responses to individual messages (Level 1) nested within participants (Level 2), and therefore models between-trial dependencies within participants. All between-participant predictors (including trait affect, biospheric and egoistic value orientations, social desirability, age, time, and gender) were mean-centered across participants so that fixed-effects coefficients could be interpreted relative to the relevant means. Positive ERBs (i.e. the dependent variable) was dummy coded as 1 for the relevant behavior or 0 otherwise (i.e. baseline). Random effects included random intercepts at the participant level and time (i.e., a variable from 1 to 50 according to signal number, which was centered around zero to allow for model convergence) was included as a random slope.

Next, we analyzed to what extent committing or being exposed to ERBs was related to participants’ current affective state, and whether individual differences in positive affect moderated this relationship. To this end, we conducted a linear mixed-effects model, where the fixed effects included each type of ERB reported (committed positive, committed negative, exposed-to positive, exposed-to negative; each predictor was effects-coded with nonERB set to the baseline), positive trait affect, age, gender, social desirability, and time (all centered as in the previous models). Random effects included random intercepts at the participant level and time as a random slope. To test the boundaries of the interactions between trait affect and positive ERBs (i.e. for both committed and exposed to positive ERBs), we additionally conducted a Johnson–Neyman simple slopes analysis (with alpha = 0.05) using the interactions package in R (see Fig. 1B,C).

### Experiment 2

Participants for Experiment 2 were recruited online between March 2018 to May 2019 from Mechanical Turk. Similar to Experiment 1, in order to be included in the study, participants were required to be at least 18 years old, have a personal smart phone with an active data plan, successfully answer 3 attention checks in the questionnaires, and respond to at least half (i.e. 6/12) of the experience sampling signals. Participants were offered a $2 compensation plus an additional $0.50 for each message they responded to (totaling $16). Unlike Experiment 1, the experience sampling protocol utilized a cellphone application survey distribution approach (via expiwell.com), wherein participants received a notification on their phone and directly completed the survey inside the application. Once they had successfully completed the intake survey (where they provided informed consent) and training, participants were randomly assigned to one of three experimental groups where they would receive the intervention message (i.e., the positive news, negative news, or non-environmental news) each morning randomly between 8 am and noon. Next, they were asked to rate the intensity to which they felt each of 8 different emotions (on a 100-point slider) including pride, joy, anger, disgust, guilt, fear, hope, and relief. The 4 positive emotions were averaged to create one composite score (Cronbach’s alpha = 0.91). Three times throughout the rest of the day, participants were asked to report an ERB that occurred within the last 1 h, using the same protocol as in Experiment 1. This research was approved by the ethics committee of the Faculty of Psychology and Educational Sciences of the University of Geneva, Switzerland and all research was performed in accordance with relevant guidelines and regulations. Written informed consent was obtained from all participants.

We first analyzed to what extent trait affect related to changes in positive affect after reading each intervention message. We then conducted a multiple linear regression model where the average positive emotion score was the dependent variable and event type (i.e., group) and trait positive affect were included as independent variables. Regression results are shown in Supplementary Table S5.

Next, we analyzed to what extent being exposed to different types of environmentally relevant information each morning interacted with trait affect to influence pro-environmental behavior throughout the rest of the day. Similar to Experiment 1, we conducted a mixed effects logistic regression analysis, which included positive ERB commission as an effects coded dependent variable, as well as type of intervention message and trait positive affect (centered across participants, alongside the other control variables) as independent variables. Random effects included random intercepts at the participant level and time as a random slope. To test the boundaries of the interactions between group and type of message, we additionally conducted a Johnson–Neyman simple slopes analysis (with alpha = 0.05) using the interactions package in R (see Fig. 2).

## Supplementary Information

Supplementary Information.
