# Supplementary material for: Interindividual differences in environmentally relevant positive trait affect impacts sustainable behavior in everyday life
Source: Sci Rep. 2021 Oct 14;11:20423. doi: 10.1038/s41598-021-99438-y (PMC8516924; doi:10.1038/s41598-021-99438-y)
Supplement: Supplementary file 1 — Supplementary Information. [file 41598_2021_99438_MOESM1_ESM.docx]

# Supplementary Materials for

**Interindividual differences in environmentally relevant positive trait affect impacts sustainable behavior in everyday life**

Kimberly C. Doell*, Beatrice Conte, Tobias Brosch

*Corresponding Author. E-mail: Kimberly.doell@unige.ch

# Experiment 1

## Methods

### Experimental Overview

During the experiment intake, all participants first followed a link to Qualtrics, an online survey platform, where they read and virtually signed a consent form, reported demographic information, and completed the questionnaires. They were then directed to SurveySignal.com, an online experience sampling (ES) platform, and were asked to verify their smartphone (see^1^). Starting from the next day, participants began the experience-sampling portion of the experiment. They would receive a text message to their phone containing a link to Qualtrics, where they would report about their daily behaviors.

### Participants

Participants for Experiment 1 were recruited online between October 2017 and April 2018 in two large-scale advertisement waves via various forums (Facebook groups, crowdsourcing and citizen science webpages, Reddit, and Amazon’s Mechanical Turk). Because this type of experience sampling for environmental behaviors had never been conducted before, alongside the difficulty associated with power calculations and various factors that contribute to power analyses in experience sampling models (see^2,3^), in Experiment 1, we heuristically chose to recruit 200 participants. However, given the fact that we had a larger than expected drop-out rate (either because they failed the attention checks or they opted to complete the online questionnaires but not the experience sampling; see below), we did not have a large enough sample size after the first phase of recruitment. Thus, we ran a second recruitment wave, which resulted in 181 participants to be analyzed. To be included in the study, participants were required to be at least 18 years old, have a personal smart phone with an active data plan, successfully answer 3 attention checks in the questionnaires, and respond to at least half (i.e. 25/50) of the ES text messages. Participants were offered a base $10 compensation, plus an additional $0.25 for each text message they responded to (maximum of $22.50 USD) in Amazon Online gift cards (or paid via MTurk). Due to technical issues, participants from China and Turkey were prevented from signing up for the experiment. Overall, 72.4% were from the United States, 10.5% from the United Kingdom, 3.9% from Italy, 3.3% from Canada, and 9.9% elsewhere.

### Questionnaires

Value Orientations: all participants responded to the long version of the Schwartz Value Scale^4^, with the addition of the supplementary biospheric value orientation questions^5^ resulting in 59 total items. Each item was rated on a scale from -1 to 7 according to -1: “Opposite to my values”, 0: “Not important at all”, 3: “Important”, 6: “Very important”, 7: “Of supreme importance”. The relevant values were calculated according to^6^. Briefly, for each participant, each item was centered by the average of the entire 59 items, and then each value was calculated from the mean of the respective 3 or 4 items.

Previous research has identified 4 particular value orientations which are thought to meaningfully contribute to positive ERB commission: biospheric, egoistic, altruistic, and hedonic^7^. However, as there was a relatively high correlation between all 4 value orientations (Table S1; all r>.28, p<.003) in order to reduce any potential confounds caused by multicollinearity, we chose not to include all 4 items in our regression models. Because we were specifically interested in understanding how biospheric and egoistic values relate to both positive and negative environmental behaviors, we focused on analyzing those 2 specific value orientations in the regression models.

Trait Affect: participants also completed the Environmental Trait Affect Questionnaire^8^, an instrument which assesses an individual’s disposition to experience affect in several types of environmentally relevant situations. Because we were specifically interested in understanding how individual’s trait emotions elicited from experiencing positive (e.g. pride, gratitude) and negative (e.g. guilt, indignation) emotions in environmentally relevant situations, related to positive and negative environmental behaviors respectively, we focused on the positive and negative outcome affects subscales. It should be noted that these two subscales represent combinations of personally committing environmental behaviors (e.g. “I feel PROUD when I act in an environmentally friendly manner”) as well as being exposed to such behaviors (e.g. “I feel DISGUSTED when others waste natural resources”). Again, these two subscales were positively correlated (r=0.61, p<0.001), so to reduce multicollinearity confounds, they were not included in the same regression models. Positive trait outcome affect was utilized to predict positive ERBs, while negative outcome affect was used to predict negative environmental behaviors.

Social Desirability: Finally, to account for social desirability bias, participants completed the 13-item social desirability scale (SDS) short form^9^. This scale is in a true-false response format. Each answer is scored and summed to create an overall continuous variable of degree of bias. The SDS score was added to all regression models alongside age and gender, as control variables.

| Table S1: Pearson correlations for all questionnaires and emotions (Experiment 2) | | | | | | | |
| --- | --- | --- | --- | --- | --- | --- | --- |
|  |  | Egoistic Values | Biospheric Values | Positive Trait Affect | Negative Trait Affect | Social Desirability (SDS) | Average Positively Valenced Emotions |
| Experiment 1 (N=181) | Biospheric Values | -.454^**^ |  |  |  |  | -- |
|  | Positive Trait Affect | -.239^**^ | .362^**^ |  |  |  | -- |
|  | Negative Trait Affect | -.242^**^ | .508^**^ | .613^**^ |  |  | -- |
|  | Social Desirability (SDS) | -0.144 | 0.057 | 0.044 | 0.082 |  | -- |
|  | Age | -.223^**^ | .191^*^ | .174^*^ | .263^**^ | 0.097 | -- |
| Experiment 2 (N=331) | Biospheric Values | -.677^**^ |  |  |  |  |  |
|  | Positive Trait Affect | -.415^**^ | .464^**^ |  |  |  |  |
|  | Negative Trait Affect | -.396^**^ | .562^**^ | .636^**^ |  |  |  |
|  | Social Desirability (SDS) | -0.087 | .165^**^ | .142^**^ | .141^*^ |  |  |
|  | Age | -0.081 | .142^**^ | 0.028 | .113^*^ | 0.084 |  |
|  | Average Positively Valenced Emotions | .108^*^ | 0.067 | .175^**^ | .139^*^ | 0.102 | 0.064 |
|  | Average Negatively Valenced Emotions | 0.020 | 0.100 | .156^**^ | .169^**^ | 0.049 | -0.036 |
|  | *p<.05; **p<0.01 (two-tailed) | | | | | | |

### ES

Training: Following the online questionnaires, participants received seven examples of different types of environmental behaviors/non-environmental behaviors and were asked to classify them as a “committed positive environmental behavior”, “committed negative environmental behavior”, “seen/read/heard about positive behavior”, “seen/read/heard about a negative behavior”, or “not environmentally relevant”. This included items such as “I recycled my plastic bottle” (i.e. a committed positive ERB), or “I went for a run” (i.e. a “none”). These examples were given to train the participants about the different categories throughout the ES phase.

ES protocol: The ES utilized an SMS survey distribution approach, wherein the participants received a hyperlink to a short survey on Qualtrics, via text message, directly to their personal smartphone. Participants were signaled 5 times per day for 10 days randomly between the hours of 9 am and 10 pm. If they did not respond, they were sent a reminder message within 15 minutes, and after 1 hour the signal expired. On each signal, they were asked to choose from one of 5 options: “I committed a positive [/negative] environmental action” (i.e. committed positive/negative), “I saw or read or heard about a positive [/negative] environmental action” (i.e. exposed to positive/negative), or “I did not commit nor learn about anything environmentally relevant” (i.e. none). Participants were then asked to briefly describe the event, and finally were asked about their state affect (i.e. mood) on an 11-point scale from very negative (i.e. 0) to very positive (i.e. 10).

Data Cleaning: 134 participants failed at least one of the 3 attention checks during experimental intake, 297 participants completed the intake questionnaires, but then opted not to participant in the experience sampling portion of the study, and 99 participants responded to less than 50% of the SMS messages (range 1:24 responses, M=9.16, SD=7.4). Consequently, 181 participants (age: M= 33.5, SE=0.88, range=18 to 76; 61% F, 0.6% unreported) were included in the current study with a mean response rate of 79.2% (i.e. M=39.6, SE=0.56). Two research assistants independently reviewed each behavior description to correct for erroneous participant classifications. Only in cases where there was an obvious error by the participant, were the classifications changed. For example, a participant reported none, but then wrote “I recycled my bottle” or a positive ERB was marked for “I forgot the lights on when I left for work”. We did not reclassify any case where it was ambiguous as to what the participant meant (e.g., “it's good” marked as committed positive) or how it was related to the environment in the context of this experiment (e.g., “My friend helped an elderly” marked as an exposed to positive). We then compared the any changes between the two RAs and any discrepancies (e.g., one flagged a description to be changed but the other did not, or one reclassified a response differently from the other) were discussed openly between the two until a consensus was reached. Overall, 374 responses were changed (i.e., 5% of messages in total).

Descriptive and Frequency Data: Overall, from the 181 participants responded with 7,161 valid responses. Of these, 2,675 (37.4 %) were non-ERBs, and 4,486 (62.6 %) were environmentally relevant. A Χ^2^ analyses revealed that the type of action (i.e. committed and witnessed) and valence of the action (i.e. positive and negative) were associated such that people were more likely to report committing a positive action (Χ^2^(1)= 70.05, p<.001, Cramer’s V=0.13).

Positive/Negative environmental behavior analyses (Fig. 1A in main paper; Table S2 & S3): We first tested whether positive trait affect was associated with the likelihood to report committing positive and negative behaviors separately. It should be noted that specific values (e.g. biospheric and egoistic values) have been shown previously to explain a large portion of variance related to positive trait affect see^8^. Thus, to any model that contained trait affect we also added biospheric and egoistic core values to control for their impact. Given that each participant completed between 25 to 50 messages, we utilized multi-level (logistic) regressions, which predicts responses on individual messages (Level 1) nested within participants (Level 2), and therefore models between-trial dependencies within participants. All between-participant predictors (e.g. value orientations, and trait affect) were mean centered across participants so that fixed-effects coefficients could be interpreted relative to the relevant means. Analyses were implemented in R, using the lme4 package (Bates *et al.*, 2014; version 1.1-21). Each dependent variable was dummy coded as 1 for the relevant behavior or 0 otherwise (i.e. baseline). Thus, we conducted mixed effects logistic regression models (with logit link). Random effects included random intercepts at the participant level and time (i.e. a variable from 1 to 50 according to signal number, which was centered around zero to allow for model convergence) was included as a random slope.

In both analyses (i.e. committed positive and committed negative), Model 1 included the fixed effects for biospheric values, egoistic values, and respective trait outcome affect. Model 2 additionally controlled for effects of age, social desirability, gender, and time. Because compared to Model 1, Model 2 was shown to have a higher marginal R^2^ (i.e. indicates the variance explained by the fixed effects alone) and conditional R^2^ (indicates the variance explained by fixed and random effects together), we report the findings from Model 2 for each analysis in the main paper and show the odds ratios from all models are shown in Table S2 and S3.

State affect analysis (Fig. 1B and C in main paper): Similar to the previous analyses, we utilized a mixed model analysis, however as state affect was a continuous variable, we employed a linear model (again in R via the lme4 package). In this case, the fixed effects included each type of behavior reported (committed positive & negative, exposed to positive & negative; each effects-coded where non-environmental behaviors [i.e. NonERB] represented the baseline), positive trait affect, age, gender, social desirability, and time (all centered as in the previous models). Random effects included random intercepts at the participant level and time.

## Results

### Committed positive ERB analysis

From the committed positive ERB analysis, we mainly expected that positive trait affect would be positively associated with the likelihood to commit positive ERBs. Positive trait affect was significant (OR=1.21, CI= 1.06 – 1.38, p=0.004; Fig. 1A in the main paper), suggesting that those that experience stronger positive emotions in environmentally relevant situations, report committing more positive ERBs. From the control variables, social desirability (OR=1.04, CI=1.00 – 1.07, p=0.048) and time (OR=0.99, CI=0.99 – 1.00, p<0.001) were both significant. This suggests a small effect of social desirability bias on reported positive ERBs, and a small decrease in reporting positive ERBs over time. Fixed effect predictors accounted for 1.9% of the variance (i.e. marginal R^2^=0.019) whereas fixed and random effects jointly explained 16.4% of the variance (i.e. conditional R^2^).

| **Table S2:** Multilevel Binomial Logistic (with logit link) Regression Models Predicting Likelihood to Commit **Positive** Environmental Behaviors (i.e. positive ERBs) in Experiment 1. | | | | | | | |
| --- | --- | --- | --- | --- | --- | --- | --- |
|  | **Model 1** | | | | **Model 2** | | |
| Predictors | Odds Ratios | | CI | p | Odds Ratios | CI | p |
| (Intercept) | 0.57 | | 0.51 – 0.65 | <0.001 | 0.53 | 0.47 – 0.60 | <0.001 |
| Biospheric Values | 1.00 | | 0.91 – 1.10 | 0.918 | 1.00 | 0.91 – 1.09 | 0.929 |
| Egoistic Values | 1.05 | | 0.93 – 1.18 | 0.413 | 1.08 | 0.96 – 1.22 | 0.190 |
| Pos. Trait Affect | 1.22 | | 1.07 – 1.38 | 0.003 | 1.21 | 1.06 – 1.38 | 0.004 |
| Social Desirability Scale |  | |  |  | 1.04 | 1.00 – 1.07 | 0.048 |
| Age |  | |  |  | 1.01 | 1.00 – 1.02 | 0.070 |
| Gender |  | |  |  | 0.96 | 0.85 – 1.07 | 0.449 |
| Time |  | |  |  | 0.99 | 0.99 – 1.00 | <0.001 |
| Random Effects | | | | | | | |
| σ^2^ | | 3.29 | | | 3.29 | | |
| τ_00_ | | 0.57 _Participant_ | | | 0.55 _Participant_ | | |
| τ_11_ | | 0.00 _Participant.Time_ | | | 0.00 _Participant. Time_ | | |
| ρ_01_ | | 0.70 _Participant_ | | | 0.82 _Participant_ | | |
| ICC | | 0.15 | | | 0.15 | | |
| N | | 181 _Participant_ | | | 180 _Participant_ | | |
| Observations | | 7161 | | | 7136 | | |
| Marginal R^2^ | | 0.008 | | | 0.019 | | |
| Conditional R^2^ | | 0.155 | | | 0.164 | | |
|  | | | | | | | |

### Committed negative ERB analysis

The analysis of the committed negative environmental behavior analysis is shown in Table S3. Negative trait affect was unrelated to negative ERB commission (OR=1.12, CI=0.97 – 1.31, p=0.121). However, biospheric value orientation approached significance (OR=0.86, CI=0.74 – 1.01, p=0.061), suggesting a possible negative relationship between biospheric values and negative ERBs. From the control variables, age was negatively associated (OR=0.96, CI=0.95 – 0.98, p<0.001), suggesting that older adults reported committing fewer negative environmental behaviors and gender was positively associated (OR=1.23, CI=1.03 – 1.48, p=0.021), suggesting that women reported more negative ERBs compared to men. Fixed effects predictors accounted for 6.6% of the variance (i.e. marginal R^2^) whereas fixed and random effects jointly explained 34.6% of the variance (i.e. conditional R^2^).

| **Table S3:** Multilevel Binomial Logistic (with logit link) Regression Models Predicting Likelihood to Commit **Negative** environmental behaviors in Experiment 1. | | | | | | | |
| --- | --- | --- | --- | --- | --- | --- | --- |
|  | **Model 1** | | | | **Model 2** | | |
| Predictors | Odds Ratios | | CI | p | Odds Ratios | CI | p |
| (Intercept) | 0.66 | | 0.54 – 0.80 | <0.001 | 0.62 | 0.51 – 0.74 | <0.001 |
| Biospheric Values | 0.85 | | 0.72 – 1.00 | 0.055 | 0.86 | 0.74 – 1.01 | 0.061 |
| Egoistic Values | 1.00 | | 0.82 – 1.21 | 0.976 | 0.95 | 0.79 – 1.15 | 0.605 |
| Negative Outcome ETA | 1.05 | | 0.90 – 1.23 | 0.548 | 1.12 | 0.97 – 1.31 | 0.121 |
| Social Desirability Scale |  | |  |  | 0.98 | 0.93 – 1.03 | 0.457 |
| Age |  | |  |  | 0.96 | 0.95 – 0.98 | <0.001 |
| Gender |  | |  |  | 1.23 | 1.03 – 1.48 | 0.021 |
| Time |  | |  |  | 0.99 | 0.99 – 1.00 | 0.064 |
| Random Effects | | | | | | | |
| σ^2^ | | 3.29 | | | 3.29 | | |
| τ_00_ | | 1.51 _Participant_ | | | 1.26 _Participant_ | | |
| τ_11_ | | 0.00 _Participant.Time_ | | | 0.00 _Participant. Time_ | | |
| ρ_01_ | | 0.18 _Participant_ | | | 0.28 _Participant_ | | |
| ICC | | 0.31 | | | 0.30 | | |
| N | | 181 _Participant_ | | | 180 _Participant_ | | |
| Observations | | 7161 | | | 7136 | | |
| Marginal R^2^ | | 0.009 | | | 0.066 | | |
| Conditional R^2^ | | 0.321 | | | 0.346 | | |
| Notes: ETA=environmental trait affect | | | | | | | |

### Affective experiences in the field

Following the report of their behavior, participants also report their current affective state. Here, we aimed to determine the effect that reporting each type of environmental behavior (compared to non-environmental behavior) had on influencing their current affective state. We hypothesized that committing or being exposed to a positive ERB would result in a more positive affective state, while committing or being exposed to a negative environmental behavior would result in a more negative affective state. In addition, we also wanted to determine how reporting a behavior influenced affective state as a function of positive trait affect. For example, we can hypothesize that committing a positive ERB results in an increase in momentary affective state, even more so if the participant has high *positive* trait affect. A similar hypothesis can be made for merely being exposed to (i.e. seeing, reading, or hearing about) a positive ERB. Congruently, we could also propose an interaction between negative behaviors and negative outcome affect, as well as exposed to negative and negative outcome affect. However, to avoid overfitting the model, and to reduce any confounds of multicollinearity, we avoided adding positive and negative outcome affect together inside of the same model. Given that we did not find an association between negative outcome affect and negative behavior commission (but we did for positive commission and positive trait outcome affect), we chose to drop the trait negative outcome affect analysis.

We again conducted a multi-level analyses, (see supplementary Table S4), utilizing a linear mixed model (fit by REML). Like the analyses reported above, we first calculated the regression model with our relevant predictors (i.e. dummy variables for committed positive and negative environmental behavior and exposed to positive and negative environmental behavior). The second model also included the control variables (social desirability, age, and gender), and the final model (reported below) also calculated the interaction between committed positive ERBs and positive trait affect, as well as exposed to positive ERB and positive trait affect. In support of our hypotheses, all main effects of the four behaviors were significant and congruent with the valence of the behavior (committed positive: b=0.59, SE=0.048, t(7002)=12.2, p < 0.001; exposed to positive: b=0.52, SE=0.087, t(6923)=5.93, p < 0.001; committed negative: b=-0.87, SE=0.061, t(6970)=-14.1, p<0.001; exposed to negative: b=-1.53, SE=0.098, t(6985)=-15.5, p < 0.001). In addition, the interaction between committed positive ERB and positive trait affect was significant (b=0.35, SE=0.046, t(6989)=7.69, p < 0.001) and the interaction between exposed to positive and trait outcome affect closely approached significance (b=0.19, SE=0.097, p=0.053). As can be seen in Fig. 2A, in both cases, as positive trait affect increased, so too did momentary affect, but the increase was steeper when reporting committing a positive ERB or being exposed to a positive ERB, compared to baseline.

Simple slopes analyses were implemented to determine where the slopes of these interactions differed from each other (see Fig. 2A in the main paper). To do so we utilized the interactions package in R and ran a Johnson Neyman’s analyses (with alpha = 0.05). For the committed positive ERB interaction, the slopes were significantly different when positive trait affect was outside the interval -2.33 to -1.24 (note, the observed range of positive trait affect values was from ‑2.87 to 1.13). This suggests that for people who were very low on trait positive trait affect, committing a positive ERB resulted in a decrease in affective state (relative to baseline). For the exposed to positive ERB interaction, the slopes were significantly different when positive trait affect was greater than -1.19, suggesting that those with those with high positive trait affect received a boost in affective state from merely being exposed to a positive ERB. It should be noted however, that for both interactions, the confidence intervals in the predicted data for those low in positive trait affect were relatively larger than those with high outcome affect. This suggests that there were fewer participants on the left side of the distribution than the left, thus decreasing the power of the interaction there.

| **Table S4:** Multilevel Linear Regression Models Predicting State Affect in Experiment 1. | | | | | | | | | |
| --- | --- | --- | --- | --- | --- | --- | --- | --- | --- |
|  | Model 1 | | | Model 2 | | | Model 3 | | |
| Predictors | Estimates | CI | p | Estimates | CI | p | Estimates | CI | p |
| (Intercept) | 6.54 | 6.35 – 6.74 | <0.001 | 6.55 | 6.37 – 6.74 | <0.001 | 6.55 | 6.36 – 6.74 | <0.001 |
| Committed positive ERB | 0.63 | 0.53 – 0.72 | <0.001 | 0.61 | 0.51 – 0.70 | <0.001 | 0.59 | 0.49 – 0.68 | <0.001 |
| Committed Negative ERB | -0.85 | -0.97 –  -0.73 | <0.001 | -0.85 | -0.97 –  -0.73 | <0.001 | -0.87 | -0.99 –  -0.75 | <0.001 |
| Exposed to positive ERB | 0.52 | 0.35 – 0.70 | <0.001 | 0.52 | 0.35 – 0.69 | <0.001 | 0.52 | 0.35 – 0.69 | <0.001 |
| Exposed to Negative | -1.62 | -1.81 –  -1.43 | <0.001 | -1.56 | -1.75 –  -1.37 | <0.001 | -1.53 | -1.72 –  -1.33 | <0.001 |
| Time | -0.08 | -0.14 –  -0.02 | 0.006 | -0.08 | -0.14 –  -0.02 | 0.007 | -0.08 | -0.14 –  -0.02 | 0.008 |
| SDS |  |  |  | 0.07 | 0.02 – 0.13 | 0.011 | 0.07 | 0.02 – 0.13 | 0.011 |
| Age |  |  |  | 0.01 | -0.01  – 0.03 | 0.205 | 0.01 | -0.01  – 0.03 | 0.209 |
| Gender |  |  |  | -0.04 | -0.23  – 0.14 | 0.650 | -0.04 | -0.22  – 0.15 | 0.691 |
| Pos. Outcome Affect |  |  |  | 0.37 | 0.18 – 0.56 | <0.001 | 0.24 | 0.04 – 0.43 | 0.019 |
| Committed positive ERB x Pos. Trait Affect |  |  |  |  |  |  | 0.35 | 0.26 – 0.44 | <0.001 |
| Exposed to positive ERB x Pos. Trait Affect |  |  |  |  |  |  | 0.19 | -0.00  – 0.38 | 0.054 |
| Random Effects | | | | | | | | | |
| σ^2^ | 2.56 | | | 2.54 | | | 2.52 | | |
| τ_00_ | 1.58 _pcpID_ | | | 1.41 _pcpID_ | | | 1.42 _pcpID_ | | |
| τ_11_ | 0.09 _pcpID.time3_ | | | 0.10 _pcpID.time_ | | | 0.09 _pcpID.time_ | | |
| ρ_01_ | 0.18 _pcpID_ | | | 0.18 _pcpID_ | | | 0.19 _pcpID_ | | |
| ICC | 0.40 | | | 0.37 | | | 0.37 | | |
| N | 181 _pcpID_ | | | 180 _pcpID_ | | | 180 _pcpID_ | | |
| Observations | 7155 | | | 7130 | | | 7130 | | |
| Marginal R^2^ | 0.087 | | | 0.137 | | | 0.142 | | |
| Conditional R^2^ | 0.448 | | | 0.458 | | | 0.463 | | |
| Notes: ERB=Environmentally relevant behavior; SDS=social desirability scale; | | | | | | | | | |

# Experiment 2

## Methods

### Experimental Overview

During the experiment intake, all participants first followed a link to Qualtrics, where they read and virtually signed a consent form, reported demographic information, and completed the questionnaires. Once they had successfully completed the intake survey, participants were randomly assigned to one of 3 experimental groups: positive environmental, negative environmental, or non-environmental. We utilized a cell-phone application-based experience sampling protocol which utilized the application Expimetrics (expimetrics.com). Following the intake, participants were provided with information on how to download and register their smartphone for the Expimetrics app. Starting from the next day, participants began the ES portion of the experiment. It should be noted that only participants utilizing either an Android or iOS phone could sign up for this experiment because of the restrictions from the app.

### Participants

To determine sample size, we conducted a conservative a priori power analysis (with 3 groups, power=0.80, alpha=.05, and a small effect size of 0.15) which suggested 432 participants in total. Given the exclusion and dropout rates from the previous experiment, we decided to over-recruit 530 participants in total. Participants from Experiment 2 were recruited online between March 2018 to May 2019. In Experiment 1, we did not acquire any identifying information, so to prevent the same participants from signing up for the Experiment 2, all participants were recruited via Amazon’s Mechanical Turk, where we were able to include/exclude workers based on their worker IDs. Thus, 95.5% of participants were from the United States, 1.8% were from Canada, and 2.7% from elsewhere. Like Experiment 1, to be included in the study, participants were required to be at least 18 years old, have a personal smart phone with an active data plan, successfully answer 3 attention checks in the questionnaires, and respond to at least half (i.e. 6/12) of the ES signals. Participants were offered a base $2 compensation, plus an additional $0.50 for each message they responded to (totaling $16).

### Questionnaires

Participants all responded to the same intake survey and questionnaires as in Experiment 1 (via Qualtrics).

### ES

Training: Participants received the same training session as in Experiment 1.

ES protocol: Each morning (between 8 am to noon), participants would receive notification on their phone (via the Expimetrics app) containing an environmental intervention message (according to their group). Three times throughout the rest of the day (i.e. noon to 10pm) participants would receive the same assessment protocol as in Experiment 1.

The intervention messages contained information about a recent news story that was either environmentally positive (e.g. how efforts to plant trees could reduce climate change), negative (e.g. statistics about climate change and its negative effects), or non-environmental (e.g. newly discovered information about snake cannibalism). Following the intervention, participants were then asked, “Please rate the intensity of each emotion you are feeling as a result of reading this information.” for each of 8 different emotions: pride, joy, anger, disgust, guilt, fear, hope, and relief. Participants indicated their intensity for each emotion on a 100-point slider scale (0= “very low intensity” to 100= “very high intensity”). We then averaged all the positive and all the negative emotions to create 2 continuous variables (i.e. average positive emotions and average negative emotions).

Data Cleaning: 34 participants failed at least one of the 3 attention checks during experimental intake and 78 participants were removed for responding to less than 50% (i.e. 6/12) of the signals (range=1:5 responses, M=2.1, SD=1.8). 14 participants were removed for not attending to the experiment’s instructions (e.g., all behavior descriptions were random character strings, or nonsensical responses). Overall, 73 participants completed the intake, but then opted not to complete the experience-sampling portion of the experiment. One independent research assistant also reviewed each behavior description to correct for erroneous participant classifications. Only in cases where there was an obvious error by the participant, were the classifications changed. For example, a participant reported none, but then wrote “oops, I did commit an environmental behavior, I recycled my bottle” or a positive ERB was marked for “I forgot the lights on when I left for work”. We did not reclassify any case where it was ambiguous as to what the participant meant (e.g. “it's good” marked as committed positive) or how it was related to the environment in the context of this experiment (e.g. “My friend helped an elderly” marked as an exposed to positive). For each response marked as erroneous, another researcher (KD) reviewed and approved of each change. In total 101 (i.e. 3.6%) of all responses were recoded. Finally, any day where a participant did not receive the intervention message, that entire day was removed from the analysis.

Descriptive and Frequency Data: Overall, from the 331 participants included, they responded with 2,203 valid responses. Of these, 925 (42%) were committed positive, 199 were committed negative (9%), 196 (8.9%) were exposed to positive, 77 (3.5%) were exposed to negative, and 806 (36.6%) were nonERBs. The Χ^2^ analyses was conducted to examine the differences between rates of reporting between the 3 groups and was found to be non-significant (Χ^2^(2) = 7.26, p<0.51).

Data analysis: the analysis of Experiment 2 contained 2 main parts. In the first we wanted to analyze how positive trait affect was linked to the ratings of the positive emotions from the intervention messages. To do so we first created an averaged daily positive emotion score (i.e. the average of pride, joy, hope, and relief; all r>0.70; non-environmental news group: M=33.00, SE=1.88; positive news group: M=64.10, SE=2.25; Negative news group: M=20.57, SE=1.55). This score was then entered as the dependent variable into a multiple linear regression model in order to predict the valence of the positive emotions experienced based on the type of environmental message received (i.e. group) as a function of positive trait affect. We again utilized the lme4 package in R and added all the relevant control variables to the analysis, as well as the interactions between group and positive trait affect (see Table S5). Group was effects coded such that non-environmental news group represented the baseline.

In the second analysis we aimed to investigate how positive trait affect interacted with group to influence positive ERB commission, we again conducted a mixed effects logistic regression analysis (Table S7). We added the same centered control variables as in the other regression analyses (i.e. gender, age, and social desirability). Group was effects coded with positive news group representing the “baseline” condition. We chose to effects code in this way because we were interested in comparing the positive news group with the non-environmental news group, as well as positive news group with negative news group. If we had chosen non-environmental news group to be the baseline (like in our other analyses), it would have resulted in the comparison between negative news group and the non-environmental news groups, predicting the likelihood to commit positive ERBs (i.e. an analysis that is theoretically uninteresting).

## Results

### Affective experiences in the field (i.e. valence of positive emotions experienced)

Table S5 and Fig. S1 show the results from the linear multiple regression model predicting valence of positive emotions felt as a function of positive trait affect. Congruent with our predictions, the interactions between positive trait affect and the emotion induction groups (i.e. positive and negative news groups) were significantly different from the control group (i.e. non-environmental news group). More specifically, compared to non-environmental group, the positive news group experienced heightened positive emotions, while the negative news group experienced decreased positive emotions, but only when positive trait affect was high (Positive news group: b=6.10, SE=2.45, t(323)=2.49, p=0.01; negative news group: b=-5.88, SE=2.71, t(323)=-2.17, p=0.031; both compared to the non-environmental news group group). Simple slopes analyses revealed that in the positive vs. non-environmental news group comparison, the slopes were significantly different when positive trait affect (z-scored) was greater than -2.91, while the slopes from the negative vs. non-environmental news comparison differed when positive trait affect was greater than -0.93. These results confirm that not only did our manipulation worked, but that the experience of positive environmental emotions in the real world, depend on your level of positive trait affect.

| **Table S5:** Linear regression results predicting strength of Positively Valenced Emotions | | | | | | |
| --- | --- | --- | --- | --- | --- | --- |
|  | **Model 1** | | | **Model 2** | | |
| Predictors | Estimates | CI | p | Estimates | CI | p |
| (Intercept) | -0.23 | -0.36 –  -0.10 | <0.001 | -0.23 | -0.35 –  -0.10 | <0.001 |
| Positive News Group | 1.19 | 1.01 – 1.37 | <0.001 | 1.2 | 1.02 –  1.38 | <0.001 |
| Negative News Group | -0.45 | -0.63 –  Ta-0.27 | <0.001 | -0.46 | -0.64 –  -0.29 | <0.001 |
| Positive Trait Affect | 0.2 | 0.06 – 0.34 | 0.005 | 0.16 | 0.02 –  0.30 | 0.022 |
| Positive News Group x Positive Trait Affect | 0.19 | 0.01 – 0.37 | 0.037 | 0.23 | 0.05 –  0.40 | 0.013 |
| Negative News Group x Positive Trait Affect | -0.24 | -0.44 –  -0.04 | 0.018 | -0.22 | -0.41 –  -0.02 | 0.031 |
| Age |  |  |  | 0.02 | -0.06 – 0.09 | 0.651 |
| SDS |  |  |  | 0.13 | 0.06 –  0.21 | 0.001 |
| Observations | 331 | | | 331 | | |
| R^2^ / R^2^ adjusted | 0.531 / 0.523 | | | 0.548 / 0.538 | | |
| Notes: SDS=Social Desirability Scale | | | | | | |

| 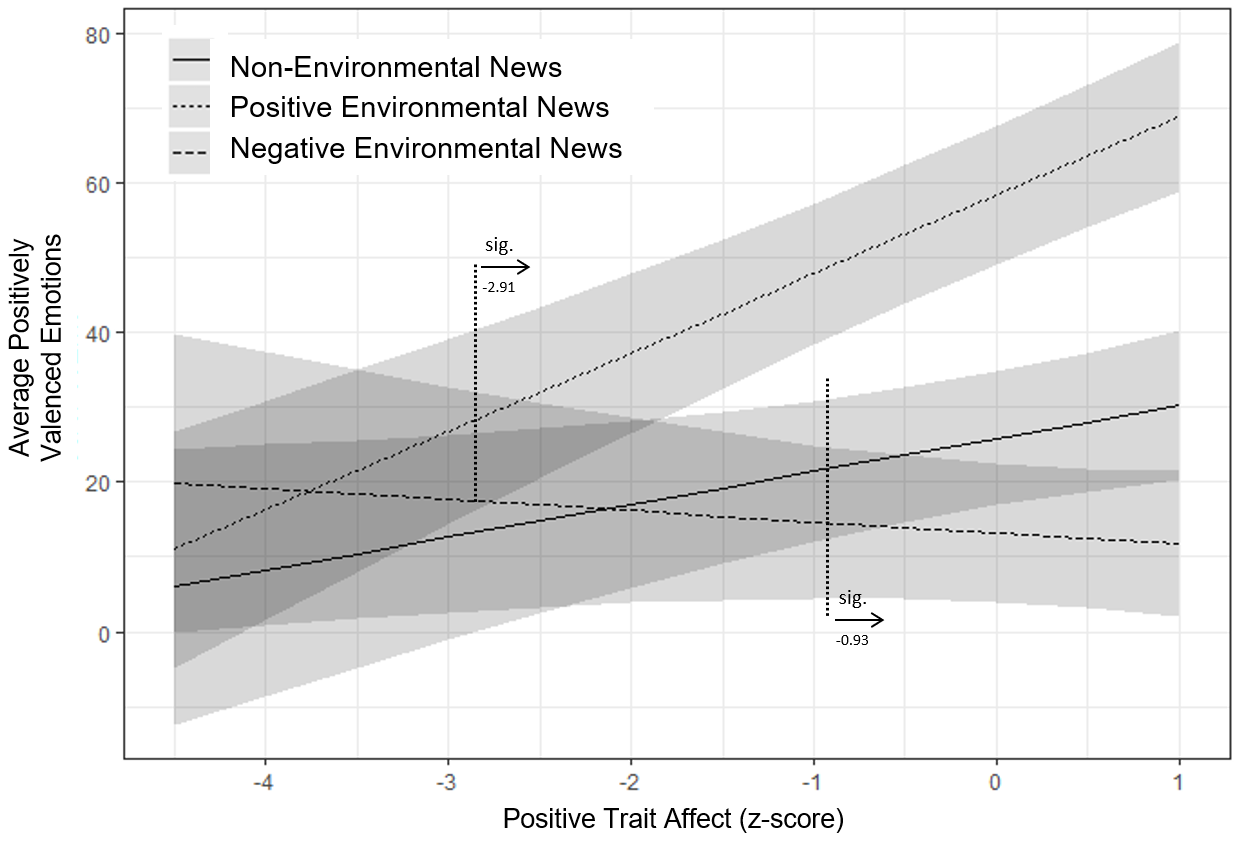  **Figure S1.** Interaction between Group and Positive Trait Affect (z-scored values) on the predicted values of the averaged positively valenced emotions experienced each morning following reading the news-style information. The vertical dotted lines illustrate where the positive news group’s slopes (left) and negative news group’s slope (right) significantly differ from the Non-environmental group, as determined by Johnson Neyman analyses (with alpha=0.05). errors=95% CIs. |
| --- |

### Committed positive ERB analysis

From the committed positive ERB analysis we expected that the group that received positive environmental messages in the morning would commit more positive ERBs throughout the rest of the day, as compared to those that received non-environmental or negative environmental messages. The results from the multilevel binomial logistic regression analysis (with logit link) revealed significant interactions between each group, and positive trait affect (Fig. 2 in the main paper and Table S6).

# A small discussion about the effect sizes in relation to our results

It should be noted that we do not necessarily interpret our effect sizes (e.g., the odds ratios) listed throughout our manuscript in terms of change in behavior nor real-world “impact”. This was an intentional decision by the authors, as we believe that doing so in the context of our results is not only difficult, but likely also futile, and misses the main take home messages that we are attempting to convey throughout our manuscript. However, we still report the odds ratios, R^2^, and other metrics of effect sizes throughout our manuscript in case others would like to translate them as needed (e.g., for meta-analyses).

First, if we were to translate the effect sizes in relation to a change in positive trait affect, because it is a personality scale, it would still lack real world significance (e.g., what would a “one-point increase” in trait affect mean in a real-world context?). Second, translating our effect sizes into percentage of increase in sustainable behavior would also lack real world meaning, as participants were free to choose any environmental behavior they wished. For example, one participant may have increased the frequency that they shut off the lights before leaving a room, while another participant may have purchased solar panels for their home (see Table 1 in the main article). We therefore cannot claim anything related to impact of the behaviors beyond stating that “participants increased their positive ERBs by X percent, compared to the other groups”.

Rather than saying “similar behavior change interventions should be deployed now”, the impact/significance of our results are twofold. First, we show that interventions/communications that rely on emotional messaging can boomerang, resulting in unintended side effects. For example, for participants that were low on trait affect, being exposed to positive environmental news results in them committing fewer positive ERBs compared to those that were shown non-environmental or negative environmental news (and there were no significant differences between the negative and non-environmental groups). Thus, inter-individual differences need to be accounted for when designing such interventions/communications (e.g., by utilizing message tailoring).

Second, our results provide further support for the positive feedback loop outlined in the discussion (see also ^11^). Anticipating feeling good after committing a positive ERB, will increase the likelihood that people engage in more positive ERBs, reinforcing that cycle. Additionally, feeling good after being exposed to the good deeds of others (i.e., vicarious warm glow), can be leveraged to kick-start this cycle (as shown in Experiment 2), potentially even reinforcing the warm glow/affective predisposition/trait affect that originally led to the first behavior. Given that the fight against climate change cannot be won by promoting one-shot environmental behaviors that occur under only specific conditions, instead we need to focus on promoting changes towards more sustainable lifestyles, which may begin with targeting such inter-individual traits like (vicarious) “green” warm glow, which will ultimately encourage a variety of positive ERBs (see the discussion in the main article for a more in-depth representation of our results).

| **Table S6:** Multilevel binomial logistic regression (with logit link) results predicting likelihood to commit positive ERBs in Experiment 2 | | | | | | | | |
| --- | --- | --- | --- | --- | --- | --- | --- | --- |
|  | **Model 1** | | | | **Model 2** | | | |
| Predictors | Odds Ratios | | CI | p | Odds Ratios | | CI | p |
| (Intercept) | 0.73 | | 0.61 – 0.88 | 0.001 | 0.72 | | 0.60 – 0.86 | <0.001 |
| Non-environmental new group | 0.88 | | 0.68 – 1.13 | 0.31 | 0.90 | | 0.71 – 1.16 | 0.429 |
| Negative news group | 1.02 | | 0.79 – 1.32 | 0.85 | 1.02 | | 0.80 – 1.31 | 0.855 |
| Positive trait affect | 1.49 | | 1.27 – 1.74 | <0.001 | 1.45 | | 1.24 – 1.69 | <0.001 |
| Non-environmental news group x positive trait affect | 0.8 | | 0.63 – 1.01 | 0.055 | 0.75 | | 0.60 – 0.95 | 0.017 |
| Negative news group x positive trait affect | 0.69 | | 0.55 – 0.87 | 0.002 | 0.67 | | 0.54 – 0.85 | 0.001 |
| Age |  | |  |  | 1.01 | | 1.00 – 1.02 | 0.066 |
| SDS |  | |  |  | 1.03 | | 1.00 – 1.06 | 0.022 |
| Gender |  | |  |  | 0.88 | | 0.79 – 0.97 | 0.013 |
| Biospheric Values |  | |  |  | 1.12 | | 0.99 – 1.26 | 0.070 |
| Egoistic Values |  | |  |  | 1.02 | | 0.91 – 1.15 | 0.739 |
| Random Effects | | | | | | | | |
| σ^2^ | | 3.29 | | | | 3.29 | | |
| τ_00_ _pcpID_ | | 0.21 | | | | 0.17 | | |
| ICC | | 0.06 | | | | 0.05 | | |
| N _pcpID_ | | 331 | | | | 328 | | |
| Observations | | 2203 | | | | 2190 | | |
| Marginal R^2^ | | 0.028 | | | | 0.040 | | |
| Conditional R^2^ | | 0.087 | | | | 0.088 | | |
| **Notes**: The grouping variables are effects coded such that the positive news group represents the “baseline”. SDS=Social Desirability Scale | | | | | | | | |

# References

1. Hofmann, W., Wisneski, D. C., Brandt, M. J. & Skitka, L. J. Morality in everyday life. *Science (80-. ).* **345**, 1340–1343 (2014).

2. Caine, K. Local Standards for Sample Size at CHI. in *Proceedings of the CHI Conference on Human Factors in Computing Systems, ACM* 981–992 (2016). doi:10.1145/2858036.2858498

3. Berkel, N. Van, Ferreira, D. & Kostakos, V. The Experience Sampling Method on Mobile Devices. *ACM Comput. Surv.* **50**, 1–40 (2017).

4. Schwartz, S. H. Universals in the content and structure of values: Theoretical advances and empirical tests in 20 countries. *Adv. Exp. Soc. Psychol.* **25**, 1–65 (1992).

5. de Groot, J. I. M. & Steg, L. Value Orientations and Environmental Beliefs in Five Countries: Validity of an Instrument to Measure Egoistic, Altruistic and Biospheric Value Orientations. *J. Cross. Cult. Psychol.* **38**, 318–332 (2007).

6. Schwartz, S. H. & Littrell, R. Draft Users Manual: Proper Use of the Schwarz Value Survey. *compiled by Romie F. Littrell.* (2009).

7. Steg, L., Perlaviciute, G., van der Werff, E. & Lurvink, J. The Significance of Hedonic Values for Environmentally Relevant Attitudes, Preferences, and Actions. *Environ. Behav.* **46**, 163–192 (2014).

8. Hahnel, U. J. J. & Brosch, T. Environmental trait affect. *J. Environ. Psychol.* **59**, 94–106 (2018).

9. Reynolds, W. M. Development of Reliable and Valid Short Forms of the Marlowe-Crowne Social Desirability Scale. *J. Clin. Psychol.* **38**, 119–126 (1982).

10. Bates, D., Mächler, M., Bolker, B. & Walker, S. Fitting Linear Mixed-Effects Models using lme4. **67**, (2014).

11. Brosch, T. Affect and emotions as drivers of climate change perception and action: a review. *Curr. Opin. Behav. Sci.* **42**, 15–21 (2021).
